# Glucose Level Sensing Using Single Asymmetric Split Ring Resonator

**DOI:** 10.3390/s21092945

**Published:** 2021-04-22

**Authors:** Gameel Saleh, Ijlal Shahrukh Ateeq, Ibraheem Al-Naib

**Affiliations:** Biomedical Engineering Department, College of Engineering, Imam Abdulrahman Bin Faisal University, Dammam 31441, Saudi Arabia; gsmohammed@iau.edu.sa (G.S.); Lsateeq@iau.edu.sa (I.S.A.)

**Keywords:** glucose level, metamaterial, asymmetric split resonator, rectangular waveguide

## Abstract

In this article, a biosensor composed of a single metamaterial asymmetric resonator is specifically designed for sensing the glucose level of 1 µL of solution. The resonator has two gaps, and one of them ends with a semicircle shape on which the glucose solution is placed. This design helps in confining the drops of glucose solutions in a specific area where the field is maximally confined in order to enhance the electromagnetic wave-matter interaction. Six samples of glucose solutions with concentrations that cover hypoglycemia, normal and hyperglycemia conditions that vary from around 41 to 312 mg/dL were prepared and examined by this biosensor. The resonance frequency redshift was used as a measure of the changes in the glucose level of the solutions. Without glucose solution, an excellent agreement between the measured and simulated transmission amplitude was observed. The increase in glucose concentrations exhibited clear and noticeable redshifts in the resonance frequency. This biosensor revealed a 0.9997 coefficient of determination, which implies an excellent prediction fitting model. More importantly, a sensitivity of 438 kHz/(mg/dL) was observed over the range of concentrations of the aqueous solution.

## 1. Introduction

According to a recent study from the World Health Organization and the international diabetes federation, there are around 463 million diabetes cases worldwide [1,2]. Generally, diabetes results when the pancreas is not able to produce enough insulin, or the latter is not effectively delivered to the cells. In turn, the normal carbohydrate metabolism is affected and leads to an increase in the glucose levels in the blood [1]. More specifically, deficient production and inefficient use of insulin cause diabetes type 1 and type 2, respectively. When the blood sugar level is less than 70 mg/dL, the situation is called hypoglycemia [2]. Conversely, it is called hyperglycemia when the blood sugar level is more than 120 or 180 mg/dL when fasting or after meals, respectively [2,3].

Glucose level monitoring is essential to avoid the many well-known complications when abnormal levels of glucose in the blood remain for a long time [4,5]. Blood glucose can be monitored using invasive, minimally invasive and noninvasive devices [6,7,8,9,10]. The invasive methods use self-monitoring blood glucose devices where finger-pricking is required [11,12]. Continuous glucose monitoring devices can be achieved using minimally invasive devices where a thin lancet is implanted subcutaneously within the interstitial fluid [13]. In the noninvasive devices, no needles or direct contact with the body are needed [8,14,15]. Different techniques have been proposed for glucose level measurements such as electrical [8], optical [16,17,18,19,20,21], thermal [22,23] and radiofrequency [24,25,26,27,28,29,30,31,32,33] sensing methods.

In [24], two open V-band waveguides enclosing water and saline solutions were utilized to measure different glucose concentrations in transmission mode in the frequency band of 50–75 GHz. The best correlation between the glucose level measurements and the transmission coefficient is in the frequency ranges of 59–64 and 69–73 GHz. Another study utilized a split rectangular resonator surrounded by an aluminum case, with one face designed to operate at 1.4 GHz, and human subjects were used for measurements [28]. Correlation coefficient and mean absolute relative difference (MARD) of 0.98 and 3.8% were achieved and used to validate the accuracy of the system. The MARD indicates the absolute error between the estimated and reference blood glucose concentrations by the candidate and comparative methods, respectively. The repeatability was validated as most of the data setpoints of the human subjects exist in Zones A and B in the Clark error grid. Moreover, using an ultra-wideband microwave sensor, the sensitivity, correctness and reliability were verified by the changes in the signal energy at 6.5 GHz of blood glucose solutions in the concentration range of 0–400 mg/dL with the step of 50 mg/dL [29]. Furthermore, an millimeter-wave glucose sensor was designed to suppress the surface and diffracted waves to enhance the sensor’s sensitivity to glucose changes [27]. Two rectangular slabs were used as absorbers to minimize the multipath transmission. They enhanced the weak signals passing through the lossy tissues and doubled the sensitivity of the sensor. In another study, three microwave sensors were used to measure different glucose concentrations in human blood plasma that added to ascorbic and lactic acids solvents [30]. The results retrieve a linear change in glucose levels. However, the sensitivities change as the rest of the concentrations in the solution vary.

Dielectric spectroscopy methods have also been proposed and found that the changes in glucose concentrations have a small effect on the dielectric properties [34,35,36]. Thus, very sensitive sensors are required with high-quality factors and well-known knowledge from multidisciplinary fields. Therefore, many scientists proposed different structures and configurations using compact metamaterial resonators in order to enhance the wave-matter interaction via high-quality factor resonances across the electromagnetic field [37,38,39,40,41,42,43,44,45]. In [37], a planar microwave sensor of honey-cell-shaped complementary split rectangular resonators was designed to monitor the blood glucose level of diabetes. A high-frequency shift resolution was achieved for samples of various glucose levels. Moreover, a wearable and tunable EM sensor was designed without any time lag and achieved a high correlation, more than 0.9, between the glucose concentrations and the human hand model properties and dimensions [38]. Furthermore, a microwave sensor with a complementary asymmetric single split resonator was designed with a high quality-factor [46]. In addition, Kumar et al. designed a microwave biosensor with high sensitivity [47]. It was achieved by the interaction of the glucose with the strength of the electric field generated from an LC resonator. Moreover, in two recent patents [48,49], a radio frequency sensor was proposed for continuous glucose monitoring using wearable multiple ultrawideband antennas. Using this approach, millimeter-wave signals are transmitted beneath the skin of the hand, and multiple receiving antennas are used to detect the backscattered signal in order to estimate the glucose molecules.

In this article, a microwave biosensor composed of a single asymmetric double split-ring resonator is introduced. The wave–matter interaction between the confined electric field within the single resonator and the glucose molecules is maximized by placing the samples at the spot of a maximum electric field. The effect of this high interaction led to a very clear shift in the resonant frequency for several glucose solutions with different concentrations. This shift can be used as a measure of the glucose concertation level. The vision of this kind of sensor is to be utilized for affordable invasive measurements and to complement the noninvasive sensors as the latter will most likely require calibration a few times over a certain period. It can also be utilized at a single frequency with a compact microwave transmitter and receiver. This will allow us to build ultra-compact invasive sensors that will still be required even with the availability of noninvasive sensors.

## 2. Methodology

**Figure 1:**
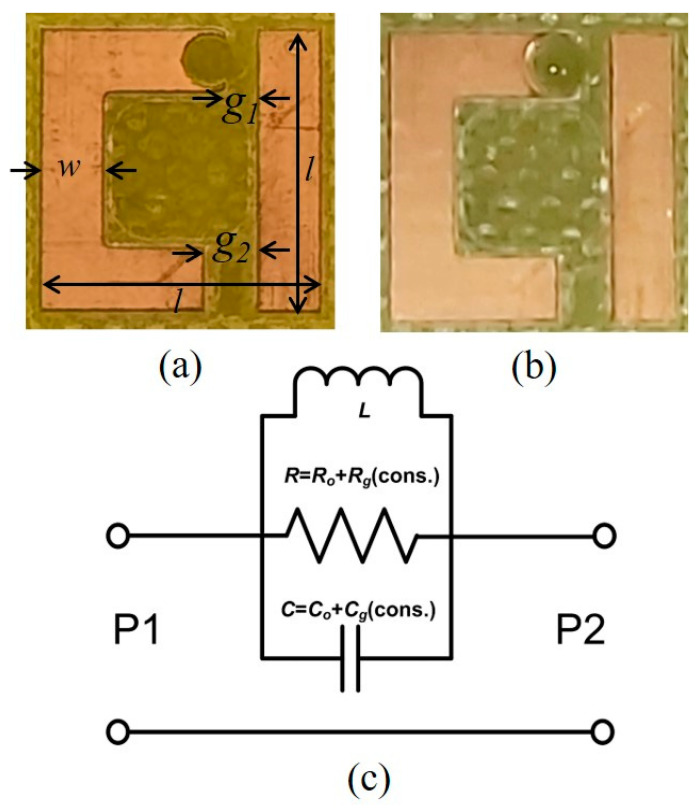
The fabricated single metamaterial asymmetric double split resonator showing the important dimensions: without the solution drop (**a**); and with the solution drop (**b**). (**c**) The equivalent circuit model with P1 and P2 representing the input and output ports, respectively.

The biosensor structure used in this study for the glucose level monitoring was adopted from asymmetric resonators that support Fano-like high-quality factor resonance that is well-known in the literature [43,50,51,52]. The working principle of these resonances is based on the coupling between narrow discrete state and broad continuum state [53]. In turn, it features a sharp asymmetric response and strong field confinement due to a significant slowdown in the speed of light due to steep phase dispersion [54]. These important characteristics support a large field–matter interaction and hence make this kind of resonances suitable for biosensing applications [55]. The fabricated design was composed of a single asymmetric split resonator (SASR), as shown in Figure 1a. The dimensions of the SASR are shown in Figure 1 with side lengths (*l*) of 7.74 mm, a width (*w*) of 1.74 mm, an upper gap (*g*_1_) of 0.925 mm and a lower gap (*g*_2_) of 1.45 mm. It was then printed by using LPKF ProtoMat S63 over FR4 substrate of 1.5 mm thickness, the permittivity of 4.1 and loss tangent tan (δ) of 0.03. The dimensions of the designed SASR are chosen to resonate at around 7 GHz, i.e., within the C-band waveguide frequency band of interests of 5.85–8.20 GHz.

Glucose is categorized under carbohydrates, and it is a simple sugar with a molecular formula C_6_H_12_O_6_ [56]_._ The footprint of the design depends on the operating frequency. In principle, the frequency of the resonator might be selected close to the molecular resonance of glucose, in order to provide a clear change in the permittivity with the change in glucose concentration, and this is roughly observed at the terahertz (THz) frequencies [34]. However, these frequencies suffer from poor penetration depth, and other effects from other molecules might be present within the same band. Hence, it might be difficult to discern small changes in the glucose concentration level [34]. In contrast, sensors working with hundred megahertz frequencies have a large wavelength and penetration depth [8]. Thus, they might be affected by the size of tissues in contact with the sensor and might require extra calibration more often. The selected frequency in our study was chosen to achieve a good penetration depth and a reasonable size of the final sensor. From an ergonomic point of view, this design at C-band would be easy to use by patients. More importantly, the price for the microwave transmitter and receiver mentioned in the vision of such a sensor would be much more affordable compared to the same equipment operating at the millimeter-wave or THz frequencies. The original design has been customized by adding a semicircle shape at the top gap to enclose the glucose solution drop, as depicted in Figure 1b. It was strategically engineered because the field is highly confined there, which should lead to high volumetric sensitivity. In the quasi-static limit, the system of the SASR inside a waveguide can be approximated by fundamental three lumped elements in parallel [36], as shown in Figure 1c. The copper part of SASR is responsible for the inductance *L*; the loss of the metal, as well as the substrate, is modeled as resistance *R* = *R_o_ + R_g_(con.)*; and the gaps can be represented by a capacitance *C = C_o_ + C_g_(con.)*, where R_o_ and C_o_ are the absolute values of *R* and *C* without any solution, respectively. When the drop of the solutions is placed at the semi-circle, the capacitance and the resistance values are increased by *R_g_* and *C_g_* modulated according to the permittivity increment at each glucose concentration (con.) level. Spectroscopy results of glucose solutions at different glucose levels have been studied previously [57,58].

**Figure 2:**
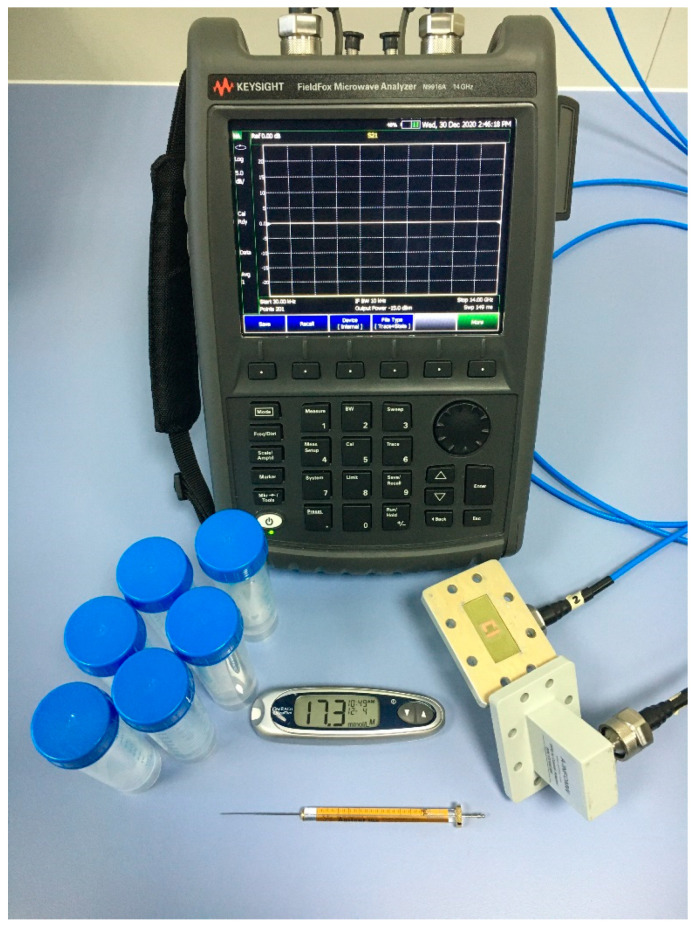
The measurement setup including the 137WCAN right-angled rectangular waveguides, the SASR structure, the samples, the syringe from Agilent model 5181-1267, the glucometer model OneTouch UltraMini and the vector network analyzer model N9916A.

The novelty aspects of this work are: (i) using a single metamaterial-based resonator instead of the conventional full two-dimensional array of resonators; (ii) sensing a tiny amount (1 µL) of a glucose solution; (iii) custom-tailored resonator with a semicircle design to confine the field and hold the solution drop on a certain spot; and (iv) using very controlled environment within a right-angled waveguide. In contrast to a full metamaterial 2D array of hundreds of resonators and free space measurements, only a single resonator is utilized in this study. Moreover, the structure is enclosed between two right-angled rectangular waveguides, as shown in Figure 2, to provide a very controlled environment and a robust shielding against the surrounding interferences. Due to the four metallic walls of the waveguide, the structure is effectively mimicking a full 2D array. The measurements were performed using a vector network analyzer model N9916A and a thru, reflect, line kit to calibrate the 137WCAN right-angled rectangular waveguides.

The transmission coefficient of the bare dielectric substrate after the calibration is recorded for the frequency band of interest and used as a reference for the normalization of all the measurements. Six different samples of glucose solutions are prepared by mixing glucose powder with distilled water in glass bottles. The weights of these compositions are measured using an ultra-sensitive electronic weighing scale and validated by using a glucometer. The prepared concentrations of the glucose samples range 41–312 mg/dL to cover hypoglycemia, normal and hyperglycemia cases in the blood of diabetic patients. Figure 2 shows the whole measurement setup including the two right-angled waveguides, the glucose samples, the fabricated structure on FR4 substrate, the glucometer that was used to measure the exact concentration of the prepared solutions, the specialized syringe that was used to take 1 µL of the solutions and the vector network analyzer.

It is worth mentioning that the design of the semicircle was essential to place the solution drops in a specific spot to ensure systematic measurement procedures and have maximum electromagnetic field–wave interaction. Replacing the semicircle with a full circle might seem more sensible but would not be effective as the field would be confined outside the circle and lead to minimal interaction with the solution. The structure was designed using the time-domain solver of the 3D electromagnetic CST Microwave Studio wave simulator. The 137WCAN waveguide with its real dimensions was considered in the dimensions, and its four electric walls were modeled using the “conducting wall” boundary conditions in the simulator. The top and bottom boundary conditions were chosen to be “open”. The waveguide ports were utilized to excite the structure. The adaptive mesh refinement was enabled along with an autoregressive filter to get converged results.

## 3. Simulated and Measured Results

**Figure 3:**
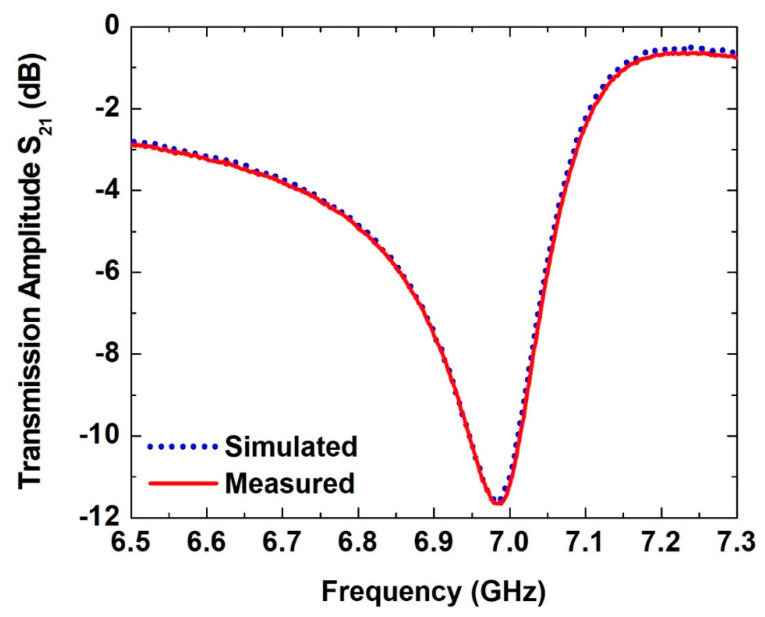
The simulated and measured transmission amplitude coefficient (S_21_) of the SASR structure without a glucose solution with reference to a bare substrate.

The normalized simulated and measured transmission amplitude coefficient (S21) of the SASR structure with respect to the bare substrate without glucose solutions is shown in Figure 3. The results show an excellent agreement between the simulated data and the measurement results. Moreover, simulating the model with *R* = 2846 Ω, *L* = 1.176 nH and *C* = 0.445 pF using the Advanced Design System software package gave very close results to the 3D simulated results. It is worth mentioning that no postprocessing or any kind of fitting method was utilized. The curves were drawn by connecting lines between the measured points. The frequency resolution in the simulated data was 1 MHz, and it was 1.875 MHz in the measured data. Furthermore, the lab temperature was controlled to be at 20 °C during the experiment. Moreover, the time required to measure each sample was less than 3 s, i.e., the time when the radio frequency signal was applied onto the sample. Given that the input power was −20 dBm and based on the earlier observation of an insignificant change in temperature due to microwave-absorption properties when heating glucose solutions at different concentrations, we estimated that the increase in the temperature of the sample was negligible [59]. The sensor was designed to resonate initially at around 7 GHz without glucose solutions. The above frequency was chosen to be close to the upper frequency of the operating frequency range of the C-band rectangular waveguides. This would give enough frequency range when the sensor is used to sense the glucose samples of 1 μL drops of different concentrations, as the resonance is expected to experience a redshift towards a lower frequency.

Although a single unit cell was used, the transmission amplitude exhibited clear resonant behavior with a quite high-quality factor due to the use of the microwave metallic waveguides for transmitting and receiving the signal. The existence of the structure inside the waveguide provided a robust shielding against surrounding interferences. For each concentration level, a drop of only 1 µL was placed on the semicircle part of the structure without any contact with the rectangular vertical to avoid a short circuit, as shown in Figure 1b. The gap between the strip and the place where the solution was confined was chosen as it provided the maximum electric field interaction with the glucose drops. Throughout the measurement procedure, careful considerations were made to maintain the measurements as consistent as possible. These included using the exact quantity of the solution for different concentrations and the exact position of the solution drop on the semicircle for all measurements. Moreover, to be precise with the concentration level, we measured them using the glucometer directly from the drops on the sensor at the same time as the measurements.

**Figure 4:**
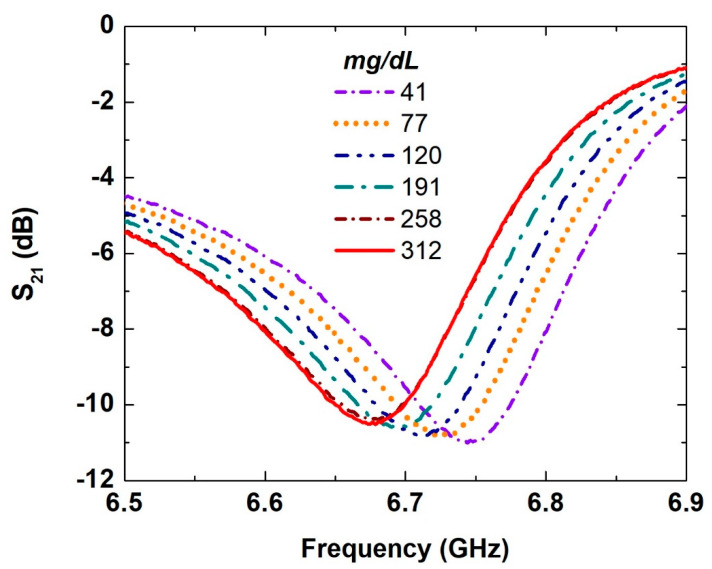
The measured transmission coefficient (S_21_) of the SASR with different glucose solutions with concentrations of 41–312 mg/dL of 1 μL drop.

The measured transmission coefficients of the proposed SASR structure with different glucose concentration levels are presented in Figure 4. Six samples of glucose solutions with different concentrations were measured. The glucose levels varied from around 41 to 312 mg/dL, as shown in the legend of Figure 4. For each glucose level, a drop of 1 μL was placed carefully on the semicircle part of the resonator. The transmission coefficient corresponding to each glucose level, with reference to a bare substrate, was measured three times to ensure data reliability, and the averaged results are plotted in Figure 4. Smooth curves without discontinuities were observed, and this was a measure of the stability of the biosensor and the reliability of the measurement setup. A clear redshift was evident in the resonance frequency with the increase in glucose concentrations. Figure 4 demonstrates that increasing the glucose concentrations could be interpreted as an increase in the capacitive reactance due to an increase in the permittivity of the sensor environment and hence shifts of the resonance to a low frequency.

The authors of [35,36,60] noticed a shift in the resonance frequency to higher frequencies compared to the resonance frequency. Moreover, the authors of [57,58] reported no change in the resonance frequency but rather a modulation in the transmission amplitude at the resonance frequency. Furthermore, the authors of [31,37,61] reported a shift in the resonance frequency towards low frequencies. Many reasons could contribute to such observations. In some of these papers, the authors used an open-ended coaxial dielectric probe to measure the permittivity of the prepared solutions with different concentrations. These measurements are quite useful but challenging at the same time, as they require special care for controlling and compensating for confounders [62]. The measurement equipment choice, measurement uncertainties and measurement calibration and validation are among many of these parameters. From a theoretical point of view, the Cole–Cole model has been utilized to calculate the complex permittivity of glucose solutions with different concentrations [63]. Some fluctuations in the infinite permittivity, the magnitude of the dispersion and the relaxation time constant can be clearly noticed for the glucose concentration levels less than 1000 mg/dL at 0.5–20 GHz band. Moreover, the exact configuration for a given sensor could also contribute to the final measured response, i.e., whether the sensor represents a simple RLC circuit or a combination of lumped elements in series and/or parallel. This suggests that further theoretical analysis of artificial glucose solutions, as well as real samples along with careful measurement procedures using different sensor configurations for a broadband frequency band with a sweep of glucose concentration with a small step are required to get the full view.

**Figure 5:**
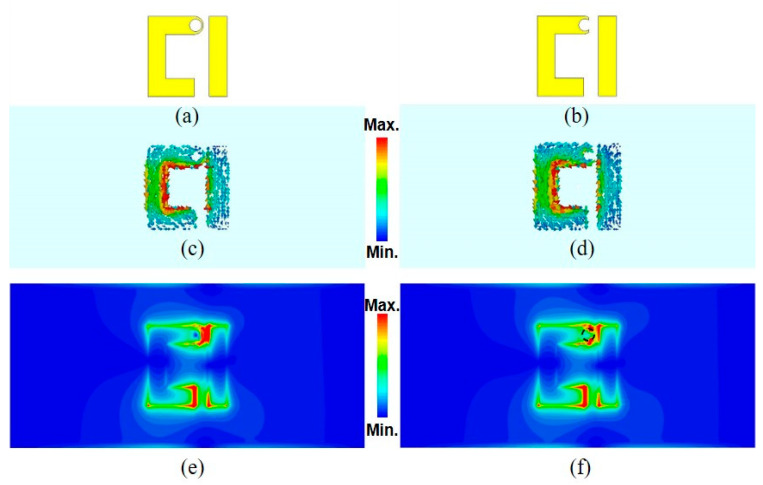
(**a**) Full-circle single asymmetric split resonator design; (**b**) semi-circle single asymmetric split resonator design used in this paper; (**c**,**d**) the surface current distribution for both designs in (**a**,**b**), respectively; and (**e**,**f**) the spatial electric-field distribution for both designs in (**a**,**b**) showing the reason behind choosing the semi-circle design and placing the glucose drops at the semi-circle spot. The dashed semicircle in (**f**) shows the spot where the glucose solution has been placed.

To explain the criteria behind using the semi-circle resonator and illustrate the field-matter interaction, Figure 5a,b presents the full and semi-circle designs, respectively. Next, the current distribution for both resonators is shown in Figure 5c,d, respectively. In both figures, an in-phase current distribution is observed, which is the main feature of the Fano-like resonance that leads to less coupling with free space and quite sharp resonance. The absolute spatial electric (|E|) field distribution of both resonators at the surface of the substrate is shown in Figure 5e,f. It might be more practical to use the full-circle design as the solution drop will be confined there. However, it would offer very low field–matter interaction as the field is confined outside the full circle, as shown in Figure 5e, and it would lead to minimal interaction with the solution. Hence, it is more rational to place the glucose sample drops on the semi-circle area exemplified here with a dashed circle, as shown in Figure 5f. As a result of high field confinement, there is maximum interaction between the glucose drops with the E-field. Increasing the glucose concentrations will accordingly modify the transmitted E-field and hence shift the resonance.

## 4. Model’s Prediction Using the Coefficient of Determination with Polynomial Fitting

**Figure 6:**
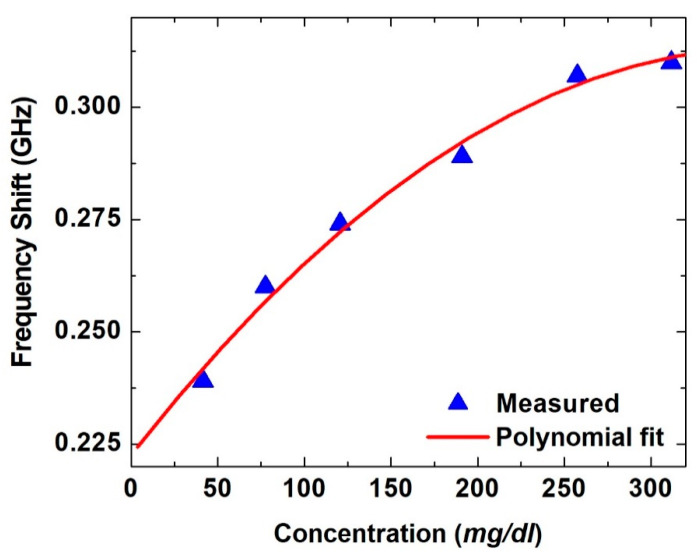
Frequency shift versus glucose concentration level for the six samples with a second-order polynomial fitting model and R^2^ = 0.997.

The resonance frequency shift at each glucose concentration is shown in Figure 6. Although the proposed SASR is composed of a single resonator, the shift in the resonance frequency is clear and noticeable. Nevertheless, it starts to saturate at concentration levels beyond 200 mg/dL. A sensitivity of 438 kHz/(mg/dL) is achieved by the proposed SASR biosensor calculated as a slop of the linear fit of the first three points where the frequency shift exhibits a linear relationship. A saturation behavior is observed with the higher concentrations, and this is in agreement with the behavior of metamaterial resonators [42]. As the frequency shift saturates at high concentrations, a nonlinear fitting curve using a second-order polynomial model, as shown in Figure 6, was used to fit the data. It could then be used to predict any glucose level corresponding to a given resonance frequency shift.

It was noticed that different criteria are used in the literature to measure the sensitivity of the introduced sensors. Therefore, it is quite difficult to hold direct comparison as it may convey unfair judgment. Some studies used the bare resonator as a reference and compared their results with others that used distilled water or aqueous solutions as a reference [64,65]. Hence, these approaches make the comparison quite difficult. Here, we relied on the measured values of the resonance shifts and hence the sensitivity was evaluated as a ratio between the change in the frequency shift to the change in the glucose level concentration. Table 1 presents a comparison of the sensitivity of the proposed sensor, some other parameters and those achieved in some previous works. Our proposed sensor exhibited a sensitivity of 438 kHz/(mg/dL) with the advantage of using a very small volume of glucose solution of 1 μL.

In [66], high sensitivity was reported; however, the quantity of the aqueous solution was not specified. Moreover, the wavelength and depth of penetration will be large at the utilized frequency range of 0.4–0.7, and the size of the tissue loaded to the sensor can affect the low-cost portable response accordingly [31]. In [67], the concentrations of the introduced aqueous solution only cover the hypoglycemia case. In the other references included in the comparison [60,64,65,68], the sensitivity is lower than the reported value in this work.

Furthermore, the coefficient of determination, denoted as R^2^, is used to show how the model can predict the data. R^2^ explains the ratio between the variance of the model’s predictions (sum of squares of residuals) to the variance of the frequency shifts from their mean (total sum of squares). This coefficient is also known as a square of the coefficient of multiple correlations, and it has a value between 0 and l, with better model fitting when it is close to 1. The polynomial coefficient of determination equation for our results exhibited an excellent R2 value of 0.9997. That means 99.9% of the variability of the frequency shifts was accounted for, and less than 0.1% of the variability was unaccounted for. It is worth mentioning that this work serves as a proof of concept and other kinds of resonators could be tested to improve the sensitivity. Likewise, other frequency bands and substrate materials could be utilized for better sensitivity.

## 5. Conclusions

In this study, a single asymmetric split resonator was successfully utilized as a biosensor to measure glucose concentration levels ranging between hypoglycemia and hyperglycemia conditions. The structure is enclosed inside two right-angled rectangular waveguides to provide strong shielding against environmental changes and interferences on the glucose solution during the measurements. For each concentration level, a drop of 1 µL was placed on the semicircle area of the sensor, and the exact value of the glucose concentration was taken from the drop on the SASR structure during the measurements. A sensitivity of 438 kHz/(mg/dL) was achieved for the tested concentration levels range. A coefficient of determination of 0.9997 was achieved by the sensor, indicating that the predicted model is quite reliable. In the future, this technique might offer another avenue for reliable glucose level measurements.

## Figures and Tables

**Table 1:** Parametric comparison of various microwave sensors for glucose level evaluation.

Ref.	Sensor Structure	Frequency Range (GHz)	Concentration (mg/dL)	Sensitivity kHz/(mg/dL)	Coefficient of Determination (R^2^)	Sensing Parameter	Sample Volume (μL)	Size (mm^3^)
[66]	A phenylboronic acid-based, hydrogel-interlayer RF resonator	0.4–0.7	0–400	304	NA	S_11_	NA	5 × 5 × 0.25
[67]	Ground-Signal-Ground *LC* resonator	1–4.5	0–72	260	NA	S_21_	NA	8 × 8 × 0.0015
[64]	Split ring resonator	1–5	0–5000	26	0.9902	S_21_	NA	50 × 20 × 1.27
[68]	complementary electric-*LC* resonator	0.8–1.8	0–10,000	21.1	0.995	S_21_	0.63	10.4 × 10.4 × 0.508
[65]	Complementary Split ring resonator	2.4–2.6	0–700	5	0.995	S_11_	70	9 × 9 × 0.764
[60]	Single port resonator	3.1–3.8	0–1000	14	NA	S_11_	125	16 × 34 × 0.813
This work	SASR	6.5–6.9	41–312	438	0.9997	S_21_	1	7.74 × 1.74 × 1.45

## Data Availability

Not applicable.
